# Polymorphisms of *HOMER1* gene are associated with piglet splay leg syndrome and one significant SNP can affect its intronic promoter activity in vitro

**DOI:** 10.1186/s12863-018-0701-0

**Published:** 2018-12-07

**Authors:** Sutong Xu, Xingjie Hao, Min Zhang, Kai Wang, Shuaifeng Li, Xing Chen, Liaohan Yang, Lin Hu, Shujun Zhang

**Affiliations:** 1 Key Lab of Breeding and Reproduction of Ministry of Education, Huazhong Agricultural University, Wuhan, 430070 Hubei China; 2 Department of Epidemiology and Biostatistics, Key Laboratory for Environment and Health, School of Public Health, Tongji Medical College, Huazhong University of Sciences and Technology, Wuhan, China; 3 Institute of Animal Husbandry and Veterinary, Wuhan Academy of Agricultural Science, Wuhan, 430208 Hubei China

**Keywords:** *HOMER1*, Piglet splay leg, Intronic promoter, SNP

## Abstract

**Background:**

In our previous genome-wide association study (GWAS) on the piglet splay leg (PSL) syndrome, the homer scaffolding protein 1 (*HOMER1*) was detected as a candidate gene. The aim of this work was to further verify the candidate gene by sequencing the gene and find the significantly associated mutation. Then we preliminarily analyzed the effect of the significant SNP on intronic promoter activity. This research provided a reference for further investigation of the pathogenesis of PSL.

**Result:**

We investigated the 19 SNPs on *HOMER1* and found 12 SNPs significant associated with PSL, including 8 SNPs resided in the potential intronic promoter region in intron 4. The − 663~ − 276 bp upstream the exon 5 had promoter activity and it could be an intronic promoter that regulated the transcription of HOMER1–205 transcript. The promoter activity of the − 663~ − 276 bp containing the rs339135425 and rs325197091 mutant alleles was significantly higher than of the wild type (*P* < 0.05). The G allele of rs325197091 (A > G) may create a new binding site of transcription factor aryl hydrocarbon receptor nuclear translocator (ARNT) and could enhance *HOMER1* intronic promoter activity.

**Conclusions:**

*HOMER1* gene was associated with the PSL, and the rs325197091 could influence *HOMER1* intronic promoter activity in vitro.

**Electronic supplementary material:**

The online version of this article (10.1186/s12863-018-0701-0) contains supplementary material, which is available to authorized users.

## Background

Piglet splay leg (PSL) syndrome is one of the complex genetic defects in newborn piglets [1]. It could be influenced by genetic and environmental factors [2]. Histomorphological and transcriptome investigations had been conducted in the normal and affected piglets, while the related genes and causal mutations are still unknown. In our initial genome-wide association study (GWAS) among 4 pig populations, we identified seven chromosome-wide significant SNPs. The most two significant SNPs, rs81360824 and rs81360870 on the chromosome 2 resided in the *HOMER1* gene (Sscrofa 10.2) [3]. *HOMER1* codes a scaffold protein and always concentrated in post-synaptic structures as well as combined with metabolic glutamate [4]. *HOMER1* was found expressed in neuromuscular junction, muscle development system and vertical sacroplasmic reticulum vesicles in human and mouse skeletal muscles [5, 6]. *HOMER1* can intact with Ca^2+^ related channel signal molecules such as IP3R, RYR, TRPC, and NFAT [7–10]. In addition, Ca^2+^/calcineurin signaling pathway is important for myotube formation, and *HOMER1* may influence the skeletal muscle function via Ca^2+^ [11]. Twelve exons and five transcriptions of *HOMER1* gene were reported in Ensemble. HOMER1–201 (ENSSSCT00000057415.1) has high homology among different species. There are nine exons that are exon 1, exon 5 to exon 12 encoded in HOMER1–201. Its translation initiation code resides on the exon 1. Other four transcripts are predicted transcript. However, HOMER1–205 (ENSSSCT00000062712.1) is associated with the skeletal muscle contraction, the development of skeletal muscle fibers, and the regulation of Ca^2+^ channel activity predicted suggested by GO analysis. Eight exons, which are from exon 5 to exon 12, are encoded in the HOMER1–205. Besides, the first 110 bp encoded in HOMER1–205 are not belonging to the coding area in other four transcripts. The translation initiation code of HOMER1–205 resides on exon 5 but the location of its 5’UTR and promoter are not clear. In this research, we aimed to characterize the polymorphism of *HOMER1* gene including the promoters of HOMER1–201 and HOMER1–205 for identifying the significant-associated mutation of PSL.

## Methods

### Animals

DNA for 183 animals, including 110 normal pigs and 73 PSL pigs from Tianzhong stock Corporation (Hubei, China), which came from the 185 animals described in our previous study [3]. We collected the ear tissues of these pigs by using ear punches as our previous study described [3].

### Cell culture

The porcine kidney cells (PK15) and the human kidney cells (293 T) were obtain from China Center for Type Culture Collection (CCTCC) and cultured using DMEM medium with high glucose (Hyclone, USA) supplemented with 10% FBS (PAN, Germany) in a humidified atmosphere of 5% CO_2_ at 37 °C.

### Semi-quantitative test of the HOMER1–205 transcript

One primer was designed and the PK15 cDNA was used to amplify the sequence of exon 5 different from other transcripts in intron 4. The forward primer resided in intron 4 while the reverse primer resided in exon 6. The primer pairs used are as follows: HM-205 T former: 5′- GTCAAGTTTGAAAGTAAGTTTCCCT -3′; reverse: 5′- AGTATTTGCCCGGCTATCGG -3′; 18srRNA former: 5′- GAGACGGTGGGACAGCG -3′; reverse: 5′- GCCCTCGGTCGAGTTGTC -3′.

### Promoter and transcription factor prediction

Bioinformatics website Neural Network Scan Service (http://www.fruitfly.org/seq_tools/promoter.html) was used to analyze the promoter regions in 3000 bp upstream the exon 1 and exon 5. JASPAR (http://jaspar.genereg.net/) were used to predict the transcription factor binding on the SNPs sites.

### SNP identification

*HOMER1* gene is located on the reverse strand. We used the reverse strand sequence as template. A total of 18 pairs of PCR primers were designed with Primer Premier 5.0 (Premier, Canada) based on the genomic sequence of the porcine *HOMER1* gene (ENSSSCG00000014113) referring to Sscorfa11.1 assembly to amplify all exons and partial adjacent introns (Additional file 1). DNA pooling strategy was used to identify potential SNPs involved in the gene. Ten DNA samples were selected to construct a DNA pool. PCRs were performed in 25 μl volume containing, 22 μl mix (TSINGKE Gold-Green mix), 1 μl of genomic DNA (50 ng/μl), 1 μl of each primer (1 μM). The PCR reaction conditions were as follows: a pre-denaturation at 98 °C for 2 min, followed by 35 cycles of 10 s at 98 °C, annealing from 50 °C to 60 °C for 10 s, 10s/kb at 72 °C, and a final extension at 16 °C for 5 min. The PCR products were sequenced using the ABI DNA analyzer (Applied Biosystems, Foster City, CA, USA). The fragments were sequenced by Sangon (Shanghai, China).

### Genotyping and association analysis

Among the identified SNPs within the region of *HOMER1*, 19 SNPs was selected according to their positions as the candidate marks to be genotyped. These SNPs were further genotyped for all experimental individuals using SNP capture on Hiseq4000 platform.

The Hardy-Weinberg test and Chisq.test in R were used to investigate the significant of those SNPs associated with PSL. *p* value < 0.05 was considered as statistically significant.

### Linkage disequilibrium (LD) measure

Haploview 4.0 was applied to analysis the haplotypes of this ten significant SNPs accorded with the Hardy-Weinberg equilibrium [12].

### Plasmid construction, transfection and dual-luciferase reporter assay

The samples with HP1 and HP2 were selected to clone the six fragments in different length. The ATG of HOMER1–205 was assigned as + 1. Six recombinant plasmids were constructed with primers shown in Additional file 2. Fragment H3W (GATC) or H3M (AGGT) covered from − 663~ + 154 bp contained wild or mutant allele of rs339135425, rs325197091, rs322755731 and rs343753765. Fragment H2W (TC) or H2M (GT) covered from − 276~ + 154 bp contained wild or mutant allele of rs322755731 and rs343753765. Fragment H1W (C) or H1M (T) covered from + 20~ + 154 bp contained wild or mutant allele of rs343753765. Those six fragments were amplified and inserted into the PGL3-Basic (Promega) between the restrictive enzyme cutting site KpnI and BgIII. Then those were transfected using lipofectamine 2000 (Invitrogen) into PK15 cells and 293 T cells. Plasmid DNA of each well used in the transfection containing 1 μg and 20 ng of the internal control vector pRL-TK Renilla/luciferase plasmid. The enzymatic activity of luciferase of the vector was measured by PerkinElmer 2030 Multilabel Reader (PerkinElmer).

### RNA interference

The sequence of double-stranded small interfering RNAs targeting ARNT is 5′-CAGACAAGCUAACCAUCUUTT-3′ (GenePharma). 2 μl of siRNA, 0.2 μg of reconstructed plasmids were co-transfected into the cells using Lipofetamine 2000™ reagent for 4 h–6 h. Then the transfection mixtures were drew out, and the complete DMEM medium was added in each well. After 24 h, the enzymatic activity of luciferase was measured by PerkinElmer 2030 Multilabel Reader (PerkinElmer).

### Western blotting

Western blotting was performed as our previously described [13]. 10–12% SDS-PAGE gels were used to separate proteins and they were transferred to polyvinylidene fluoride (PVDF) membranes (Bio-Rad). The membranes were blocked with 5% nonfat milk for 1 h and incubated with ARNT (Santa Cruz) primary antibody at 4 °C overnight. Membranes were washed with TBST (Tris - buffered saline containing 0.1% Tween 20) and incubated with HRP-conjugated secondary antibody (Proteintect) for 2 h. The relative expression normalized β-actin (Proteintect) was calculated in Image J software (NIH).

### Statistical analysis

All experiments were performed independently three times, and data were presented as the means ± standard (SEM). Data were analyzed by two-tailed Student’s t-tests with SPSS version 16.0 (SPSS, Chicago, IL, USA). A *p* value < 0.05 was considered as statistically significant.

## Result

### Preliminary detection of the 5’UTR of HOMER1–205

**Fig. 1:**
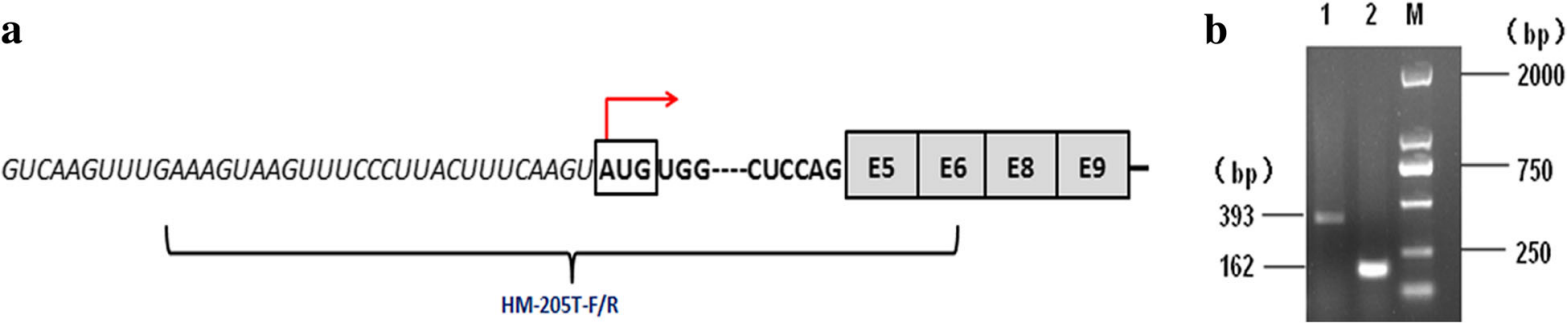
Detecting the 5′UTR of HOMER1–205 transcript. **a** The primers designed for detecting the 5’UTR of HOMER1–205 transcript. The Italic represents the intron 4 region. The bold represents the different sequence in exon 5 of HOMER1–205 transcript compared with other transcripts. **b** Lane M: Marker DL2000, Lane 1: HM-205T-F/R (393 bp), Lane 2: 18srRNA-F/R (162 bp)

The 5’UTR of HOMER1–201 is known, however, which of HOMER1–205 has not been reported. Semi-quantitative was employed to clone part of the *HOMER1* mRNA according to the sequence of HOMER1–205. One primer was designed and PK15 cDNA was used to amplify the sequence of exon 5 different from other transcripts in intron 4 including the ATG of HOMER1–205 which was the first three bases of exon 5 (Fig. 1). By sequencing, we acquired the sequence in intron 4 that may be belong to the 5’UTR upstream the ATG of HOMER1–205 (Fig. 1). The ATG of HOMER1–205 was assigned as + 1. The − 670~ − 621 bp and the − 255~ − 208 bp were predicted as promoter region by Neural Network Scan Service (Additional file 3).

### Analysis of SNPs

Six regions upstream 3000 bp of exon 1 were predicted as promoter of HOMER1–201 (Additional file 4). After sequencing twelve exons, the 1613 bp upstream the exon 1 and the 663 bp upstream the exon 5 which contained the predicted promoter regions, 21 SNPs were found (Table 1). Nineteen SNPs were genotyped. Twelve significant SNPs were identified. Eight significant SNPs (rs339135425, rs344370846, rs335396960, rs325197091, rs342088137, rs333077091, rs332280474, rs322755731) resided within the region upstream the exon 5 of 663 bp in intron 4. rs325197091 was the most significant SNP (*P* = 3.0321E-05). One SNP (rs331422651) resided in exon 5. One significant splicing mutation (rs344145865), resided in the intron 11 near the splicing site. Two significant SNPs (rs711466420 and rs709236548) were found in exon 12 belonged to the 3’UTR in HOMER1–201. Besides rs333077091 and rs709236548, other significant SNPs were in Hardy-Weinberg equilibrium (Table 1).

**Table 1:** The allele genotypes, frequencies and significance of SNPs discovered

ID	Name	Region	Position on genomic	SNP Site (Reverse stand)	Allele	Allele Freq. (Normal pigs/Affect pigs)	HW-test	Chi-square test (*P*-Value)
SNP1	rs339135425	Intron4	Chr2:88207019	G > A	G	0.72/0.52	0.4566	0.001223^**^
					A	0.28/0.48		
SNP2	rs344370846	Intron4	Chr2:88206832	G > A	G	0.87/0.77	0.4212	0.02992^*^
					A	0.13/0.23		
SNP3	rs335396960	Intron4	Chr2:88206806	C > T	C	0.88/0.76	0.4212	0.006143^**^
					T	0.12/0.24		
SNP4	rs325197091	Intron4	Chr2:88206745	A > G	A	0.51/0.29	0.05079	3.032E-05^***^
					G	0.49/0.71		
SNP5	rs342088137	Intron4	Chr2:88206700	C > T	C	0.88/0.76	0.4212	0.006143^**^
					T	0.12/0.24		
SNP6	rs333077091	Intron4	Chr2:88206655	T > C	T	0.81/0.67	0.0002	0.004711^**^
					C	0.19/0.33		
SNP7	rs323497886	Intron4	Chr2:88206523	G > A	G	0.71/0.60	0.749	0.4179^ns^
					A	0.29/0.40		
SNP8	rs332280474	Intron4	Chr2:88206451	C > T	C	0.50/0.75	0.05749	3.83E-03^**^
					T	0.50/0.25		
SNP9	rs322755731	Intron4	Chr2:88206438	T > G	T	0.47/0.27	0.2829	0.00013^***^
					G	0.53/0.73		
SNP10	rs343753765	Intron4/exon5	Chr2:88206361	C > T	C	0.63/0.54	0.7639	0.1004^ns^
					T	0.37/0.46		
SNP11	rs331422651	Intron4/exon5	Chr2:88206349	C > T	C	0.88/0.76	0.7409	0.005049^**^
					T	0.12/0.24		
SNP12	rs318637436	exon5	Chr2:88206180	C > T	C	0.87/0.77	0.5831	0.08725
					T	0.13/0.23		
SNP13	rs344145865	Intron11	Chr2:88144031	G > T	G	0.48/0.30	0.2249	0.000729^**^
					T	0.51/0.70		
SNP14	rs793766633	exon12/3’UTR	Chr2:88125722	- > A/AA/AAA	–	0.64/0.56	2.00E-04	0.1589^ns^
					A	0.36/0.44		
SNP15	rs792865901	exon12/3’UTR	Chr2:88125522	- > TT/TTT/TTTT/TTTTT/TTTTTTT	–	0.58/0.57	1.00E-04	0.8211^ns^
					T	0.42/0.43		
SNP16	rs711466420	exon12/3’UTR	Chr2:88124963	- > T	–	0.19/0.06	1	0.001152^***^
					T	0.81/0.94		
SNP17	rs701289492	exon12/3’UTR	Chr2:88124822	A > G	A	0.93/0.95	0.501	0.6271^ns^
					G	0.07/0.05		
SNP18	rs698648923	exon12/3’UTR	Chr2:88124516	T > C	T	0.93/0.95	0.5341	0.6069^ns^
						0.07/0.05		
SNP19	rs709236548	exon12/3’UTR	Chr2:88123927	- > AG	–	0.63/0.42	1.60E-07	0.0001241^***^
					AG	0.37/0.58		
SNP20	rs336678612	exon12/3’UTR	Chr2:88123776	G > A	/	/	/	/
SNP21	rs326987887	exon12/3’UTR	Chr2:88123705	C > T	/	/	/	/

The ID and position of all the SNPs were referred to Sscrofa11.1. HW-test was the Hardy-Weinberg test. ns *P* > 0.05;*P < 0.05;**P < 0.01;***P < 0.001. Reverse strand sequence was used

### Linkage disequilibrium (LD) analysis between the significant SNPs

**Fig. 2:**
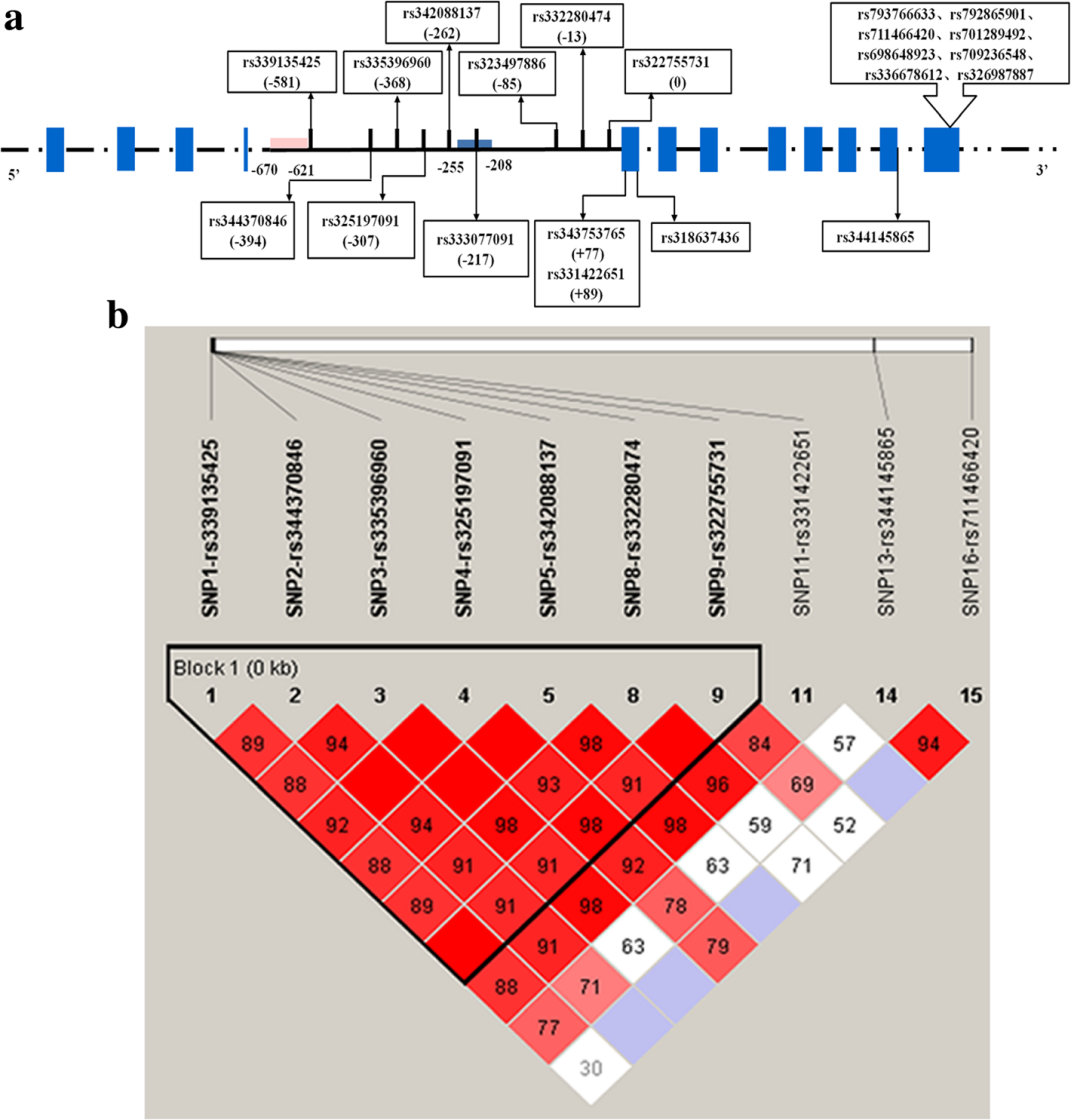
Identification of genetics varieties and the linkage disequilibrium of SNPs in porcine *HOMER1* gene. **a** Schematic diagram of the variants found in *HOMER1* gene. The blue boxes represent the exon. The dotted represent the omitted base. The number in bracket represents the position of the SNP. The number of ATG in exon 5 is assigned + 1. The two regions from -670 bp to -621 bp and -255 bp to -208 bp are the promoter region predicted by NNPP. **b** The haplotype block linkage disequilibrium value (*D’*) for 10 significant different SNPs. The values within boxes are the linkage disequilibrium value (*D’*). The darker shanding indicates higher linkage disequilibrium value

rs339135425, rs344370846, rs335396960, rs325197091, rs342088137, rs332280474 and rs322755731, those 7 significant SNPs in intron 4 composed the block1 which had five haplotypes (D’ = 1.00) (Fig. 2). The frequent of HP1 (GGCACCT) and HP2 (AGCGCCG) were 38.5% and 39.6% which were much higher than others (Additional file 5). In addition, HP1 (*P* < 0.001) and HP2 (*P* < 0.01) were significantly different between the PSL and normal pigs. HP1 contained the mutant type of rs339135425, rs325197091 and rs322755731 while HP2 contained the wild.

### Identification of *HOMER1* intronic promoter in intron 4

To detect the promoter region in intron 4 and identify whether the SNPs inside can influence its activity. The samples with HP1 and HP2 were selected to clone six fragments in different length. The ATG of HOMER1–205 was assigned as + 1. Those six constructed vectors were transfected to PK15 and 293 T cells. After 24 h, the promoter activity was detected by the Dul-Luciferase Reporter Assay System (Promega).

**Fig. 3:**
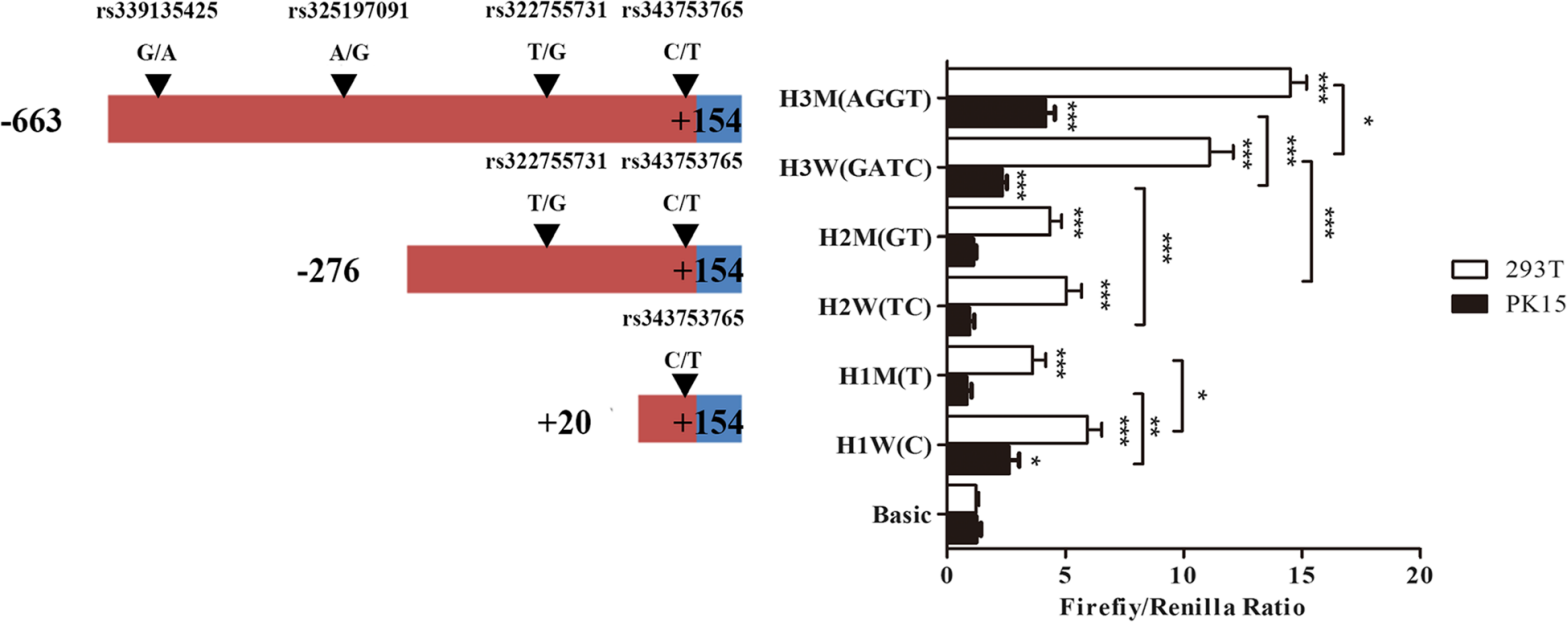
Luciferase assays of truncated vectors containing different haplotypes included SNPs of intron 4 predicted promoter. Left of the figure are the position of the fragments inserted in genome, and the number of ATG of transcript-205 was assigned + 1; Right of the figure is the relative fluorescence activity of vectors in PK15 cell and 293 T cells, Basic vector was as negative control and Firefly/Renilla Ratio indicated the relative fluorescence activity. (Mean ± SE, *n* = 9, T-test, *P < 0.05; ***P < 0.001)

Both in PK15 and 293 T cells, the promoter activity of fragment H3W (− 663 bp to + 154 bp) was significantly higher than the fragment H2W (− 276 bp to + 154 bp) (*P* < 0.05) (Fig. 3). This area also contained the rs339135425 and rs325197091, and the activity of the mutant plasmid H3M (AGGT) was significantly higher than the wild plasmid H3W (GATC) (Fig. 3).

### Interference of ARNT

**Fig. 4:**
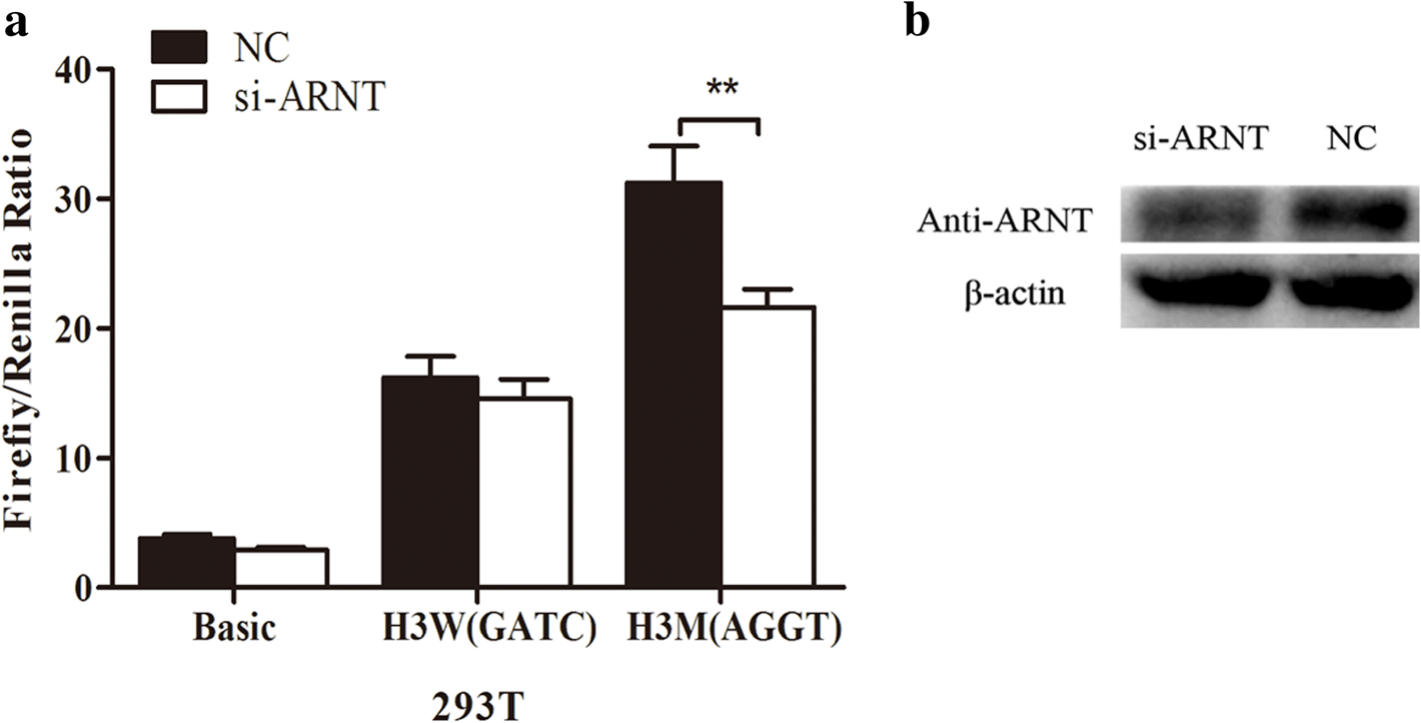
Luciferase assays of H3W (GATC) and H3M (AGGT) with interfering OCT-1 in 293 T cells. **a** The relative fluorescence activity of Basic, H3W (GATC), and H3M (AGGT) in 293 T cells with interfering ARNT. (Mean ± SE, T-test, *n* = 9, **P < 0.01) (**b**) Detection of ARNT interfered level by Western blotting

The mutation G allele of rs325197091 created the binding site of ARNT predicted by JASPAR (http://jaspar.genereg.net/) (Additional file 6). Si-ARNT was transfected with the H3W (GATC) and H3M (AGGT) into 293 T cells. The protein level of ARNT was tested by Western blotting in 293 T cells after it was interfered (Fig. 4). The activity of H3M (AGGT) containing allele G of rs325197091 was significantly decreased (*P* < 0.01) when the ARNT in lower expression while the H3W (GATC) containing the allele A was no obvious changed (Fig. 4).

## Discussion

Five transcripts were reported in Ensemble while only the HOMER1–201 transcript has been verified. However, HOMER1–205 transcript is associated with the skeletal muscle development predicted by GO analysis. As the formation of the PSL may be due to the dysplasia of muscle fibers during embryonic development [14], we just selected HOMER1–201 and HOMER1–205 as our research objects. Promoter is a key element for regulating transcription. So we detected the SNP on the predicted promoter region of HOMER1–201 and HOMER1–205.

The 5’UTR of HOMER1–201 is part of intron 1. Its promoter must be located upstream the exon 1. The 5’UTR of HOMER1–205 had not been reported. Its ATG are the first three bases of exon 5 shown in Ensemble. In addition, the first 110 bp coded in exon 5 of HOMER1–205 were not coded in other four transcripts. Then we designed a pair of primer to amplify this specific sequence in porcine PK15 cells. Particularly, the follow primer was located in intron 4. We got the sequence containing part of intron 4 and the specific 110 bp fragment as result shown. Alternative promoter is located in intron that drive the expression of alternative RNA isoforms that contain downstream exons [15]. Recent study identified 110 specific alternative isoforms drove by alternative intronic promoters in embryonic stem cells [16]. Various disease-related genes have alternative intronic promoter. Two regions containing the promoter elements were found predicted by NNPP in 3000 bp upstream the exon 5. Thus we assumed the promoter of HOMER1–205 was in intron 4.

As well, we used the NNPP to predict the promoter element in 3000 bp upstream the exon 1. Then, we detected the SNPs in the predicted promoter region of HOMER1–201 and HOMER1–205 and all the exons among our experiment group. We selected 19 SNPs to genotyped. After correlation analysis, 12 SNPs were found associated with the PSL. Eight significant SNPs were in the predicted promoter region in intron 4, one of which was not in the Hardy-Weinberg equilibrium. The remaining seven SNPs were rs339135425, rs344370846, rs335396960, rs325197091, rs342088137, rs332280474 and rs322755731, which composed a block. HP1 (GGCACCT) and HP2 (AGCGCCG) were two haplotypes associated with the PSL. The rs339135425, rs325197091 and rs322755731 were the different SNPs between those two haplotypes. In the meanwhile, rs325197091 was the most significant SNP and resided behind one of the predicted promoter regions in intron 4. So we suggest that rs325197091 may regulate the intronic promoter activity and then affect the expression of HOMER1–205.

Therefore, a series of truncated intronic promoter luciferase reporter vectors were constructed and identified. The promoter activity of -663~ + 154 bp upstream was significantly higher than − 276~ + 154 bp (*P* < 0.001). Hence, an independent promoter was suggested in the − 663~ − 276 bp. In addition, the promoter activity of the − 663~ − 276 bp containing the rs339135425 and rs325197091 mutant alleles was significantly higher than of the wild type (*P* < 0.05).

ARNT was the transcription factor predicted binding on rs325197091. The promoter activity of − 663~ + 154 bp mutant type was reduced (*P* < 0.01) by interfering ARNT while of the wild type was not significantly changed. Thus the mutation of rs325197091 might enhance the HOMER1–205 expression through binding ARNT in vitro. ARNT is the beta subtype of hypoxia-inducible factor (HIF-1), belonging to the basic-Helix-Loop-Helix (bHLH) – PSA (Per-Amt-Sim) family of proteins involved in cell differentiation and glycolysis which is highly expresses in skeletal muscle tissue. It can interact with HIF-1α and participates in solution and other physiological or biochemical pathways [17–19]. The cause of the PSL might relate to the abnormal glycogen metabolism [14]. Compared to the normal pigs, muscle fiber reduction and glycogen accumulation were observed in the longitudinal and transvers section of PSL pigs’ muscle tissues [20]. The distribution of glucose-6-phosphatase’s metabolites in longissimus dorsi and biceps femoris muscle were significant differences between PSL and normal pigs [20]. Although there is no direct evidence that ARNT involved in regulating the glucose metabolism, it can form a heterodimer mixture with HIF-1α and HIF-2α which could affect the skeletal muscle type and glycogen metabolism [21]. However, it is still necessary to collect individuals carrying rs325197091 to detect the expression level of HOMER1–205 between the wild and mutant. And further study should be taken to analysis the regulation net of the ARNT and *HOMER1* in PSL.

## Conclusion

In this study, we identified twelve significant SNPs associated with the PSL in *HOMER1* gene. rs325197091 is the most significant SNP and it was in a potential promoter region from − 663 to -276 bp upstream exon 5. We initially suggested rs325197091 mutation G allele may create an ARNT binding site and participate in the gene transcription regulation. However, further study is necessary to confirm the involving of ARNT in *HOMER1* gene regulation and investigate the relationship between the rs325197091 and *HOMER1* expression in PSL pigs.

## Additional files

Additional file 1: Primers for gene Polymorphism detection. A table of all the primers’ sequences, Tm, and product size using for detecting polymorphisms in *HOMER1* gene. (DOCX 18 kb)

Additional file 2: Primers for vector construction. A table of all the primers’ sequence, Tm and the lengths of fragments inserted using for vector construction, as well as the vectors’ name. The italics are the protect base, and the double marked are the enzyme locus. (DOCX 16 kb)

Additional file 3: Pig *HOMER1* gene intronic promoter predicted by Neural Network Promoter Prediction. A table of all the promoter elements’ locations and sequences resided on the region upstream the exon 5 of HOMER1–205 transcript predicted by Neural Network Promoter Prediction. The ATG of HOMER1–205 was assigned as + 1. (DOCX 16 kb)

Additional file 4: Prediction of pig *HOMER1* gene promoter at 5′ end region by Neural Network Promoter. A table of all the promoter elements’ locations and sequences resided on the region upstream the exon 1 of HOMER1–201 predicted by Neural Network Promoter Prediction. The ATG of HOMER1–201 was assigned as + 1. (DOCX 16 kb)

Additional file 5: The frequencies of haplotypes of Block1. A table of all the haplotypes’ frequencies in block and the *P*-value associated with the PSL. (DOCX 16 kb)

Additional file 6: Transcription factors binding on the allele of rs325197091 predicted by JASPAR. A table of all the predicted transcription factors’ names and target sequences binding on the wild or mutant allele of rs325197091. (DOCX 16 kb)

## Abbreviations

GWAS: Genome-wide association study
PSL: Piglet splay leg
SNP: Single nucleotide polymorphism
