# Supplementary material for: Polymorphisms of HOMER1 gene are associated with piglet splay leg syndrome and one significant SNP can affect its intronic promoter activity in vitro
Source: BMC Genet. 2018 Dec 7;19:110. doi: 10.1186/s12863-018-0701-0 (PMC6286600; doi:10.1186/s12863-018-0701-0)
Supplement: Supplementary file 1 — Primers for gene Polymorphism detection. A table of all the primers’ sequences, Tm, and product size using for detecting polymorphisms in HOMER1 gene. (DOCX 18 kb) [file 12863_2018_701_MOESM1_ESM.docx]

**Additional file1 Primers for gene polymorphism detection**

| Primer | Sequence(5'-3') | Tm(℃) | Product Size(bp) |
| --- | --- | --- | --- |
| HM-1P-1F | ATCACCCGCTTGACACTTCG | 63 | 1406 |
| HM-1P-1R | TCTCCCGAGGAGAACACACG |  |  |
| HM-exon1-F | TTCATTCTTTGCCGCTGGGA | 63 | 1034 |
| HM-exon1-R | ACAGACAATCCACCACCACC |  |  |
| HM-exon2-F | TCAGGGCTCCAAGGCATTTA | 60 | 581 |
| HM-exon2-R | AATAGCCCCAGCTCGAACAT |  |  |
| HM-exon3-F | AGGGCTGCACCTTTAAATTCC | 60.9 | 868 |
| HM-exon3-R | CAGCATGTTCGACGAGAGCA |  |  |
| HM-exon4-F | GGACTTCTATTCCCTGTATC | 50 | 554 |
| HM-exon4-R | TTCCAAGAGTAAAACTGCTA |  |  |
| HM-5'P2-1-F | TGGGCTACTTGGCTACCT | 59.1 | 1028 |
| HM-5'P2-1-R | GTGTTTGGGTCAATCTGG |  |  |
| HM-5'P2-2-F | TGTTCTATAAAGGCACCACC | 62.6 | 819 |
| HM-5'P2-2-R | TCAATCTGGAAGACATGAGC |  |  |
| HM-exon5-F | TGGCTGCTTTCAGTCTCTCG | 60 | 424 |
| HM-exon5-R | ACACTATTTTGGCATGCGACTC |  |  |
| HM-exon6-F | ACGTTTTGGCTCAGTTGGAGT | 56 | 288 |
| HM-exon6-R | TGACTTGAAGATGCAGATTTCCAC |  |  |
| HM-exon7-F | ACTAATTGCGCTCCCAACCTT | 60 | 546 |
| HM-exon7-R | CTATGTCCACTTTGGAACTCACC |  |  |
| HM-exon8-F | TCCAAAATAAGCCTACATCAGGGT | 60 | 357 |
| HM-exon8-R | TGACCTGGATTTTTCACTAGGGT |  |  |
| HM-exon9-F | TGGAAGCATCCTCATGTGTCTC | 60 | 666 |
| HM-exon9-R | AAGCTCCAGAAGACGTCCAT |  |  |
| HM-exon10-F | CCCATTATAGGGCCTCTCTGC | 60 | 523 |
| HM-exon10-R | ACTGCATCCTAAAAACATCCCA |  |  |
| HM-exon11-F | CCACCAAGTTGATGTGTTGCT | 60 | 352 |
| HM-exon11-R | AGGGAACGACTGATGCGTTT |  |  |
| HM-exon12-1F | TGGATTTCTCAGATGGCACA | 63.4 | 430 |
| HM-exon12-1R | GCCCAAACAGTCCTATGAAGAG |  |  |
| HM-exon12-2-F | GCCAAGCTACTAGAACGC | 56.4 | 1397 |
| HM-exon12-2-R | AAGAACCCTCCTCGACAT |  |  |
| HM-exon12-3-F | TCGCAATAACCTGAAGACAC | 55 | 550 |
| HM-exon12-3-R | AGACAGGGCTTTCCTCACTA |  |  |
| HM-exon12-4-F | GCCGTGTACTCTGTCTGGGTC | 65 | 1540 |
| HM-exon12-4-R | GTCCACATGTTCAGGCTATAGTCC |  |  |
