# Supplementary material for: Polymorphisms of HOMER1 gene are associated with piglet splay leg syndrome and one significant SNP can affect its intronic promoter activity in vitro
Source: BMC Genet. 2018 Dec 7;19:110. doi: 10.1186/s12863-018-0701-0 (PMC6286600; doi:10.1186/s12863-018-0701-0)
Supplement: Supplementary file 2 — Primers for vector construction. A table of all the primers’ sequence, Tm and the lengths of fragments inserted using for vector construction, as well as the vectors’ name. The italics are the protect base, and the double marked are the enzyme locus. (DOCX 16 kb) [file 12863_2018_701_MOESM2_ESM.docx]

**Additional file 2 Primers for vector construction**

| Primer | Sequence(5'-3') | Tm(℃) | Length(bp) | Vector |
| --- | --- | --- | --- | --- |
| HM-5'P(Z1)-F | *GG*GGTACCTGTTCTATAAAGGCACCACC | 61.6 | 819 | H3W/M |
| HM-5'P(Z)-R | *GA*AGATCTTCAATCTGGAAGACATGAGC |  |  |  |
| HM-5'P(Z2)-F | *GG*GGTACCACAGGACACTTGTTCATACC | 61.6 | 432 | H2W/M |
| HM-5'P(Z3)-F | *GG*GGTACCTGGAATCATCCTCATGGTTT | 61.6 | 134 | H1W/M |

The italics are the protect base, and the double marked are the enzyme locus.
