# Supplementary material for: Polymorphisms of HOMER1 gene are associated with piglet splay leg syndrome and one significant SNP can affect its intronic promoter activity in vitro
Source: BMC Genet. 2018 Dec 7;19:110. doi: 10.1186/s12863-018-0701-0 (PMC6286600; doi:10.1186/s12863-018-0701-0)
Supplement: Supplementary file 3 — Pig HOMER1 gene intronic promoter predicted by Neural Network Promoter Prediction. A table of all the promoter elements’ locations and sequences resided on the region upstream the exon 5 of HOMER1–205 transcript predicted by Neural Network Promoter Prediction. The ATG of HOMER1–205 was assigned as + 1. (DOCX 16 kb) [file 12863_2018_701_MOESM3_ESM.docx]

**Additional file 3 Pig *HOMER1* gene intronic promoter predicted by Neural Network Promoter Prediction**

| Start | End | Score | Promoter Sequence |
| --- | --- | --- | --- |
| -670 | -621 | 0.98 | GGTTGTGTTCTATAAAGGCACCACCAACACTAAATTAGTG**A**GTATCGAAC |
| -255 | -208 | 0.96 | AGAGAGCTGATATAAGAAGGCAGTGTTGCCTTGATCAATC**T**CTGGTGGAC |

The ATG in exon 5 of HOMER1-205 transcript was assigned as +1.
