# Supplementary material for: Polymorphisms of HOMER1 gene are associated with piglet splay leg syndrome and one significant SNP can affect its intronic promoter activity in vitro
Source: BMC Genet. 2018 Dec 7;19:110. doi: 10.1186/s12863-018-0701-0 (PMC6286600; doi:10.1186/s12863-018-0701-0)
Supplement: Supplementary file 4 — Prediction of pig HOMER1 gene promoter at 5′ end region by Neural Network Promoter. A table of all the promoter elements’ locations and sequences resided on the region upstream the exon 1 of HOMER1–201 predicted by Neural Network Promoter Prediction. The ATG of HOMER1–201 was assigned as + 1. (DOCX 16 kb) [file 12863_2018_701_MOESM4_ESM.docx]

**Additional file 4 Prediction of pig *HOMER1* gene promoter at 5’ end region by Neural Network Promoter**

| Start | End | Score | Promoter Sequence |
| --- | --- | --- | --- |
| -2628 | -2578 | 0.93 | ACAAAAAGAATATAAATGTGCTTGGGTTAATAGTTTAAAA**A**AGAACTGGT |
| -2603 | -2553 | 0.95 | GTTAATAGTTTAAAAAAGAACTGGTGGTCTTTCTTATTTT**C**AATTTCCAT |
| -1841 | -1791 | 0.98 | GACGTTGCTATTTAAAGGTCCTCCTGCGGGGAGGATGGAG**A**CACAGCGCG |
| -1752 | -1695 | 0.84 | GGCCAGGAGCCAGCAGAGGAGCAGAGAGCACAGCCCGCCC**G**CCGCTGGCC |
| -1684 | -1634 | 0.96 | GGGGGGAAGGAAAAAAGGACGGACAGCCGAATCTGCCGGC**C**GGCCTAGCT |
| -2040 | -2090 | 0.81 | GAAGGGCAGCCGCGAATGAAGGGCCGGGCGCGAGCCGGGC**T**TCATTGTGC |

The ATG in exon 1 of HOMER1-201 transcript was assigned as +1.
