# Supplementary material for: Polymorphisms of HOMER1 gene are associated with piglet splay leg syndrome and one significant SNP can affect its intronic promoter activity in vitro
Source: BMC Genet. 2018 Dec 7;19:110. doi: 10.1186/s12863-018-0701-0 (PMC6286600; doi:10.1186/s12863-018-0701-0)
Supplement: Supplementary file 5 — The frequencies of haplotypes of Block1. A table of all the haplotypes’ frequencies in block and the P-value associated with the PSL. (DOCX 16 kb) [file 12863_2018_701_MOESM5_ESM.docx]

**Additional file 5 The frequencies of haplotypes of Block1**

| Name | Haplotype | Frequency | Frequency | | P-Value |
| --- | --- | --- | --- | --- | --- |
|  |  |  | Affect pigs | Normal pigs |  |
| HP1 | GGCACCT | 0.385 | 0.271 | 0.459 | 3.00E-04^***^ |
| HP2 | AGCGCCG | 0.396 | 0.472 | 0.345 | 0.0156^*^ |
| HP3 | GATGTTG | 0.148 | 0.201 | 0.114 | 0.0213^*^ |
| HP4 | GGCACCG | 0.022 | 0.014 | 0.027 | 0.3944^ns^ |
| HP5 | AGCACCG | 0.011 | 0.007 | 0.014 | 0.5493^ns^ |
