# Supplementary material for: Polymorphisms of HOMER1 gene are associated with piglet splay leg syndrome and one significant SNP can affect its intronic promoter activity in vitro
Source: BMC Genet. 2018 Dec 7;19:110. doi: 10.1186/s12863-018-0701-0 (PMC6286600; doi:10.1186/s12863-018-0701-0)
Supplement: Supplementary file 6 — Transcription factors binding on the allele of rs325197091 predicted by JASPAR. A table of all the predicted transcription factors’ names and target sequences binding on the wild or mutant allele of rs325197091. (DOCX 16 kb) [file 12863_2018_701_MOESM6_ESM.docx]

**Additional file 6 Transcription factors binding on the allele of rs325197091 predicted by JASPAR**

| Allele | TFs | Sequence | Score |
| --- | --- | --- | --- |
| A | HFH-2/HFH-3/ HNF-3beta | GCACAAACATGC | 9.18/9.32/7.025 |
| G | [USF](http://consite.genereg.net/cgi-bin/jaspartf?ID=MA0093&score1=5.702&pos1=689&seq1=HM-M&name=USF&jobID=14695259147926)/[n-MYC](http://consite.genereg.net/cgi-bin/jaspartf?ID=MA0104&score1=5.992&pos1=690&seq1=HM-M&name=n-MYC&jobID=14695259147926) | AAACGTG | 5.702/5.992 |
|  | [ARNT](http://consite.genereg.net/cgi-bin/jaspartf?ID=MA0004&score1=6.112&pos1=690&seq1=HM-M&name=ARNT&jobID=14695259147926)/[ARNT](http://consite.genereg.net/cgi-bin/jaspartf?ID=MA0004&score1=8.609&pos1=690&seq1=HM-M&name=ARNT&jobID=14695259147926) | AACGTG | 6.112/8.609 |
